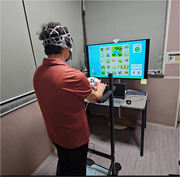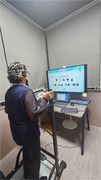# Vertification of Clinical Effectiveness of Dual‐task Cognitive Training Program and Clinical tDCS system

**DOI:** 10.1002/alz70858_101745

**Published:** 2025-12-25

**Authors:** Jong‐Hyeon Kim, Sanghun Nam, Ji‐Hyuk Park

**Affiliations:** ^1^ Yonsei University, Gangwon state, Wonju, Korea, Republic of (South); ^2^ Yonsei New‐normal Lfiestyle Resarch Center, Yonsei University, Wonju, Gangwon‐do, Korea, Republic of (South); ^3^ Yonsei University, Wonju, Gangwon‐do, Korea, Republic of (South)

## Abstract

**Background:**

The rapid aging of the global population presents significant challenges to cognitive health, leading to an increasing societal and economic burden due to rising dementia prevalence. Korea implemented its initial dementia management plan in 2008 and expanded it in 2020. However, community‐level resources remain insufficient to address the growing demand. Digital therapeutic interventions utilizing contactless technology are garnering attention, with dual‐task training and transcranial direct current stimulation (tDCS) emerging as promising approaches to cognitive enhancement. This study aimed to evaluate the combined effects of these interventions on cognitive function and dementia prevention in healthy individuals, providing evidence for their synergistic potential and contribution to early prevention strategies.

**Method:**

Participants underwent 30‐minute weekly sessions for 8 weeks, with assessments conducted during the initial and final sessions. Both groups used tDCS targeting the dorsolateral prefrontal cortex; however, only the experimental group received 1mA of stimulation. The dual‐task training comprised problem‐solving, inhibition, and working memory tasks, concurrent with stepping on a footpad. Task difficulty was incrementally adjusted and rest intervals were implemented to mitigate fatigue. Statistical analyses were performed using SPSS version 27.0, with statistical significance set at *p* <0.05.

**Result:**

Thirteen participants (1 male, 12 females; mean age 77.54±6.72 years) were included in the study. The experimental group exhibited significant improvement in CIST (pre: 20.71±8.16, post: 24.14±7.08; z=‐2.20, *p* < .05) and K‐MOCA (pre: 18.14±9.62, post: 21.14±9.28; z=‐2.21, *p* < .05), whereas no significant changes were observed in GDS, K‐IADL, and AQS (*p* > .05). The control group showed no significant changes in any of the measures (*p* > .05). A significant between‐group difference was observed in CIST scores (experimental: 3.43±2.37, control: ‐0.17±2.32; z=‐2.22, *p* < .05); however, no significant differences were noted for other measures (*p* > .05).

**Conclusion:**

The results indicate that the combination of dual‐task training and transcranial direct current stimulation (tDCS) demonstrated efficacy in enhancing cognitive function among older adults, particularly as evidenced by the CIST scores. The statistically significant improvements observed in the experimental group suggest the potential applicability of interventions that target cognitive decline. Nevertheless, the lack of significant changes in other measures necessitates further investigation to substantiate their overall effectiveness.